# Ginsenoside Rb2 Alleviates Obesity by Activation of Brown Fat and Induction of Browning of White Fat

**DOI:** 10.3389/fendo.2019.00153

**Published:** 2019-03-15

**Authors:** Yilian Hong, Yi Lin, Qiya Si, Lijuan Yang, Weisong Dong, Xuejiang Gu

**Affiliations:** ^1^Department of Endocrine and Metabolic Diseases, First Affiliated Hospital of Wenzhou Medical University, Wenzhou, China; ^2^Department of Pathology, First Affiliated Hospital of Wenzhou Medical University, Wenzhou, China

**Keywords:** Ginsenoside Rb2, obesity, browning, UCP1, AMPK

## Abstract

Ginsenoside Rb2 (Rb2), the most abundant saponin contained in Panax ginseng, has been used to treat variety of metabolic diseases. However, its effects in obesity and potential mechanisms are not well-understood. In the present study, we investigated metabolic performance with a Rb2 supplement in diet-induced obese (DIO) mice, focusing on the effects and mechanisms of Rb2 on brown and beige fat functions. Our results demonstrated that Rb2 effectively reduced body weight, improved insulin sensitivity, as well as induced energy expenditure in DIO mice. Histological and gene analysis revealed that Rb2 induced activation of brown fat and browning of white fat by reducing lipid droplets, stimulating uncoupling protein 1 (UCP1) staining, and increasing expression of thermogenic and mitochondrial genes, which could be recapitulated in 3T3-L1, C3H10T1/2, and primary adipocytes. In addition, Rb2 induced phosphorylation of AMP-activated protein kinase (AMPK) both *in vitro* and *in vivo*. These effects were shown to be dependent on AMPK since its inhibitor blocked Rb2 from inducing expressions of Pgc1α and Ucp1. Overall, the present study revealed that Rb2 activated brown fat and induced browning of white fat, which increased energy expenditure and thermogenesis, and consequently ameliorated obesity and metabolic disorders. These suggest that Rb2 holds promise in treating obesity.

## Introduction

Obesity, manifested as excessive fat accumulation, has become a global epidemic disorder that contributes to the development of several chronic diseases, including diabetes, cardiovascular diseases, and metabolic syndrome (1). As the central player in energy homeostasis, adipose tissues could be divided into the following subsets: white adipose tissue (WAT) which is characterized by large unilocular lipid-droplets-containing and functions as an active endocrine organ to regulate diverse activities, such as insulin sensitivity and brown adipose tissue (BAT) which dissipates energy as heat via the uncoupling protein 1 (UCP1) (2). Recently, a newly defined type of white adipocytes, called brite or beige adipocytes, has been shown to be reprogrammed to be brown-like adipocytes under cold or β-agonists and exhibit similar energy-consuming characteristic of brown adipocytes, which is known as the process of “browning” (3, 4). Numerous studies have suggested that genetic and pharmacological activation of brown and beige fat in mice led to enhancement of energy expenditure, thermogenesis and leanness (5). In addition to the direct regulation of energy homeostasis, brown and beige fats also function as metabolic sinks to modulate glucose and lipid metabolism, independent of their effects on weight loss (6). Importantly, these functional brown and beige adipocytes are found to exist in adults while obese and aging individuals exhibit functional defects in these adipocytes (7–9), suggesting the importance of targeting brown and beige adipocytes for treating obesity and metabolic disorders.

With the plasticity of beige adipocytes to enhance energy expenditure and thermogenesis under stimuli, massive efforts have been made to look for potent inducers for the browning effects of white fat. Apart from a few promising candidates such as fibroblast growth factor 21 (FGF21) and irisin (10, 11), active ingredients from traditional Chinese medicine (TCM) provide a wide spectrum of choices and show great potential. For example, independent studies have shown that compounds such as berberine, resveratrol, artemisinin, and celastrol coming from TCM could enhance the function of brown fat and induce browning of WAT via the transcriptional and post-transcriptional regulation of critical metabolic regulatory molecular nodes such as AMP-activated protein kinase (AMPK), silent information regulator 1 (SIRT1), peroxisome proliferator-activated receptor gamma coactivator-1a (PGC1a), and heat shock factor 1 (HSF1) (12).

Ginseng plants have been used as an ancient medicine in China for thousands of years to strengthen holistic health. As major pharmacological ingredients in ginseng plants, ginsenosides possess multiple pharmacological properties, including anti-oxidative, anti-aging, anti-cancer, and other health-improving actions (13). For metabolic aspects, research showed that ginsenosides reduced body weight (14), prevented hepatic steatosis (15), increased insulin sensitivity (16), restored mitochondrial dynamics (17), and attenuated hepatic glucagon response (18). As the most abundant saponin contained in Panax ginseng (19), Ginsenoside Rb2 (Rb2) has been reported to alleviate hepatic lipid accumulation in high-fat diet (HFD)-induced obese mice (20), decrease glycaemia in streptozotocin-induced diabetic rats (21), and lower triacylglycerol levels in 3T3-L1 adipocytes (22). We recently showed that Rb2 reduced adiposity and improved insulin sensitivity in obese mice via phosphorylation of AKT and inhibited NF-κB signaling pathway in white adipose tissues and 3T3-L1 adipocytes (23). However, the effects and mechanisms by which Rb2 alleviates obesity have not been fully elucidated, especially regarding the effects of Rb2 on brown and beige fat.

In the present study, we investigated the metabolic performances with Rb2 supplement in diet-induced obese (DIO) mice and demonstrated that Rb2 effectively reduced body weight, improved insulin sensitivity, and increased energy expenditure in DIO mice. Detailed analysis demonstrated that Rb2 induced activation of brown fat and browning of white fat as shown by reducing lipid droplets, increasing UCP1 staining and increasing brown gene programs, which could be recapitulated *in vitro*. In addition, Rb2 induced AMPK phosphorylation while AMPK activation is dispensable for the beneficial effects of Rb2. Overall, the present study revealed that Rb2 ameliorated obesity and metabolic disorders by activating brown fat and inducing browning of white fat, leading to increase in energy expenditure and thermogenesis. It was suggested that Rb2 was a promising beneficial compound treating obesity.

## Materials and Methods

### Chemicals and Antibodies

Ginsenoside Rb2 (purity >98.0%) was purchased from Shanghai Yuanye Biotech Co, Ltd (Shanghai, MO, China). The AMPK inhibitor compound C (10 mM; purity = 99.67%) was from Selleck Chemicals (S7306). Mice HFD (60% kcal from lard; MD12033) and sucrose matched low-fat control diet (10% kcal from lard; MD12031) were obtained from Research Diets. Dulbecco's Modified Eagle's Medium (DMEM), Dulbecco's Modified Eagle Media: Nutrient Mixture F-12(DMEM/F12) Medium and Fetal Bovine Serum (FBS) were obtained from Sigma-Aldrich (San José, CA, USA). Insulin powder, T3 powder, clostridium histolyticum type II collagenase, indomethacin, isobutyl-1-methylxanthine (IBMX), and dexamethasone were obtained from Sigma-Aldrich (Saint Louis, MO, USA). Primary antibodies (anti-AMPK and anti-p-AMPK) were purchased from Cell Signaling Technology (Beverly, MA, USA).

### Animals

All C57BL/6J mice (male, 8-weeks old) were housed under a constant 12 h light/dark cycle with free access to water and standard chow or HFD for 9 weeks. The mice were obtained from Shanghai Slake Experimental Animal CO.LTD., and were maintained at 22 ± 2°C with 60 ± 5% relative humidity. The HFD and chow diet-fed mice were administrated an intraperitoneal injection of Rb2 (40 mg/kg/day) or PBS (vehicle) between 16:00 and 17:00 daily. Body weight was monitored once a week between week 9 and once a day over a 10-day course of treatment. Tissues and serum were collected, snap-frozen in liquid nitrogen, and stored at −80°C. BAT was separated from the interscapular region; eWAT (epididymal WAT) was obtained from the epididymis; iWAT (subcutaneous WAT) was dissected from the layer under the skin and outside the abdominal cavity at the hips. Fat mass was the sum of BAT, eWAT, and iWAT. The experiments were randomized. All animal experiments were approved by the Institutional Animal Care and Use Committee in Wenzhou Medical University (No: SYXK-2015-0009).

### 3T3-L1, C3H10T1/2 Adipocytes, and Mice Primary Stromal-Vascular Fraction (SVF) Culture and Differentiation

3T3-L1 and C3H10T1/2 cells were purchased from the American Type Culture Collection (ATCC, Manassas, VA, USA) and were maintained in DMEM with 10% FBS mixture at 37°C in a 5% CO_2_ environment. Mice SVF was isolated from the iWAT of male C57BL/6J mice aged 6–8 weeks. SVF isolation was performed previously (24, 25). Briefly, tissues were sheared, digested with 1 mg/ml collagenase in PBS supplemented with 0.5% bovine serum albumin (BSA), and 10 mM Hepes mixture for 30 min, with 300 rpm shaking at 37°C. The suspension was strained through a 40 μM nylon mesh (Falcon), collected by centrifugation. Finally, the adipocytes were then cultured in DMEM/F12 containing 10% FBS mixture and placed inside an incubator at 37°C with a 5% CO_2_ environment.

After reaching 100% confluence, 3T3-L1, C3H10T1/2 cells, and mice SVF were stimulated to differentiate a hormone cocktail containing 50 nM insulin, 100 nM T3, 0.125 mM indomethacin, 2 μg/ml dexamethasone, and 0.5 mM IBMX. After 48 h, the adipocytes were moved to the medium containing 50 nM insulin and 1 nM T3. The media was replaced every 2 days before Rb2 treatment. Dimethyl sulfoxide (DMSO) was used as the vehicle treatment. Finally, the mature adipocytes were confirmed by light microscopy and Oil Red O staining, and then used for further analysis.

### Oil Red O Staining

Lipid droplets present within the adipocytes were proven by Oil Red O staining (Sigma). Oil Red O was diluted into water to make a 60% working solution, and then filtered through a filter paper. Adipocytes were fixed in 4% neutral buffered formalin for 30 min at room temperature. The Oil Red O solution was added (1.5 mL) and maintained at room temperature for 15 min. The solution was refused, and the wells were washed with PBS several times until the background was clear. Finally, the adipocytes were imaged using light microscopy.

### Glucose Tolerance Test (GTT) and Insulin Tolerance Test (ITT)

Previous methods were followed to implement GTT and ITT. GTT was performed in male C57BL/6J mice after an overnight fast. Blood glucose concentrations were measured from the tail vein immediately at 0, 15, 30, 45, 60, 90, and 120 min after a 0.75 g/kg i.p. injection of glucose (7) was administered. Food was removed for 2 h before the ITT was performed. After a 0.75 U/kg i.p. injection of human insulin (Eli Lilly) was administered to the mice, glucose concentrations were assessed at 0, 15, 30, 45, and 60 min (26).

### Energy Expenditure Analysis

Energy expenditure was determined using Comprehensive Lab Animal Monitoring System (CLAMS system, Columbus Instruments) according to the manufacturer's instructions. The animals were acclimated to the system for 24 h before measurement of V_O2_ and V_CO2_. The mice were maintained at 22°C under a 12 h light/dark cycle. Food and water were available *ad libitum*. Heat production was calculated according to the following equation:

$$\begin{array}{l} \text{Heat}=\left[3.815+1.232\left({\text{V}}_{\text{CO}2}/{\text{V}}_{\text{O}2}\right)\right]\times {\text{V}}_{\text{O}2}\times \text{body weight} \end{array}$$

where heat is measured in kcal h^−1^, V_O2_, and V_CO2_ are measured in liters kg^−1^ h^−1^ and body weight is measured in kg (27).

### Histological Analysis and Immunofluorescence

Tissues fixed in 4% paraformaldehyde were sectioned after being paraffin embedded. Multiple sections were prepared and stained with hematoxylin and eosin for general morphological observations. Immunofluorescence staining was performed according to standard protocols using the following antibodies and dilutions: UCP1 (Abcam) and 1:400, respectively. The incubations were performed overnight in a humidified chamber at 4°C. The secondary antibodies for immunofluorescence staining were purchased from Invitrogen. Hematoxylin staining was used to mark cell nuclei. Immunofluorescence staining was performed first, and immunofluorescent images were captured. Finally, the images were acquired using an Olympus BX51 system. And Image J was used to calculate the adipocytes size.

### Cold Tolerance Test

At the 10th day after PBS or Rb2 treatment, the mice were then subjected to a cold room (4°C) for 24 h without access to food or water. The rectal temperature of the mice was measured at 0, 30, 60, and 120 min begin at cold exposure with Th5 Thermalert Monitoring Thermometer (Braintree, US). The histological and molecular analyses were performed after the 24 h cold exposure.

### Western Blot Analysis

Adipocytes were harvested in protein extraction solution (Solarbio) and incubated for 30 min at 4°C. After cell debris was removed, supernatant containing proteins were collected and determined by using the Pierce BCA protein assay kit according to manufacturer instructions. Next, 40 μg proteins were separated by 10% SDS-PAGE and transferred to PVDF membranes. Blots were incubated by using 5% milk blocking solution at room temperature overnight with a primary antibody against AMPK and p-AMPK. The blots were then washed with TBST and incubated with the horseradish peroxidase-conjugated secondary antibody (1:5000) for an hour. Finally, the blots were developed using an enhanced chemiluminescence kit (Salarbio) according to manufacturer protocols.

### Quantitative Realtime PCR Analysis

Total RNA was isolated from cells by using Trizol (Invitrogen, Carlsbad, CA) and was reverse transcribed to first-strand cDNA using the Reverse Transcription System (A3500, Promega, Madison, WI). To analyze gene expression, real-time quantitative PCR was conducted by using an ABI Prism 7300 instrument (Applied Biosystems, Foster City, CA). Primers were provided upon requests.

### Statistical Analysis

Data were expressed as the mean ± SEM from at least three independent experiments. Analyses were performed by using Graph Pad Prism 5 and SPSS version 20.0. For repeated measurements (for example, body weight), Dunnett's multiple comparisons test were performed. For single time-point measurement, statistical analyses were performed by using an unpaired Student's *t*-test for two groups and a one-way analysis of variance (ANOVA) for more than two groups. *P* ≤ 0.05 was considered statistically significant.

## Results

### Rb2 Treatment Reduced Body Weight and Improved Insulin Sensitivity in DIO Mice

**Figure 1:**
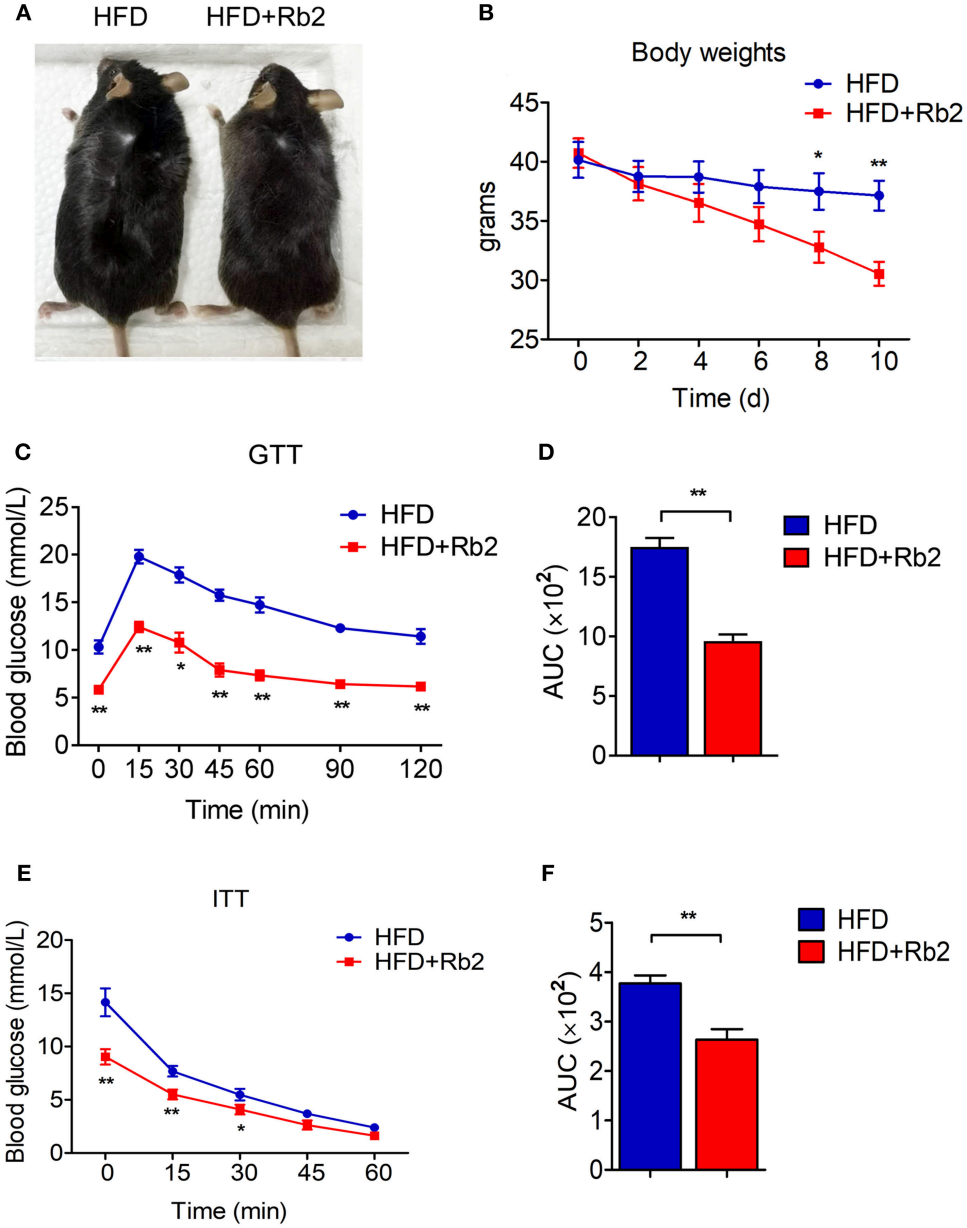
Rb2 treatment reduced body weight and improved insulin sensitivity in DIO mice. **(A)** Representative photograph of DIO mice after intraperitoneal injection of PBS or Rb2 for 10 days. **(B)** Body weight of DIO mice treated with or without Rb2 for 10 days (*n* = 6). **(C–F)** Performances of GTT **(C)** and ITT **(E)** of DIO mice treated with or without Rb2. Area under the curve (AUC) of GTT and ITT was also shown as D and F. *N* = 6 per group. Data are presented as mean ± SEM and ^*^P < 0.05, ^**^P < 0.01 compared to HFD group.

To assess the effects of Rb2 in treating obesity, we firstly established a DIO mice model by feeding mice for 9 weeks. This led to a significant increase in body weight to around 40 grams compared with chow feeding (Figures S1A,S1B). Importantly, 10 days of Rb2 treatment largely decreased body weight in these DIO mice, while not affecting body weight in mice fed by chow diet (Figures 1A,B, Figure S1C). The food intake in mice treated with Rb2 showed a slight downtrend but no significant differences when compared to the control group (Figure S1D). Meanwhile, we found that DIO mice supplemented with Rb2 had better tolerance to glucose load and were more sensitive to insulin addition, which were shown in GTT and ITT analyses (Figures 1C–F, Figures S1E–S1H). Therefore, these results demonstrated that Rb2 could reduce body weight and improve insulin sensitivity in DIO mice.

### Rb2 Treatment Increased Energy Expenditure in DIO Mice

**Figure 2:**
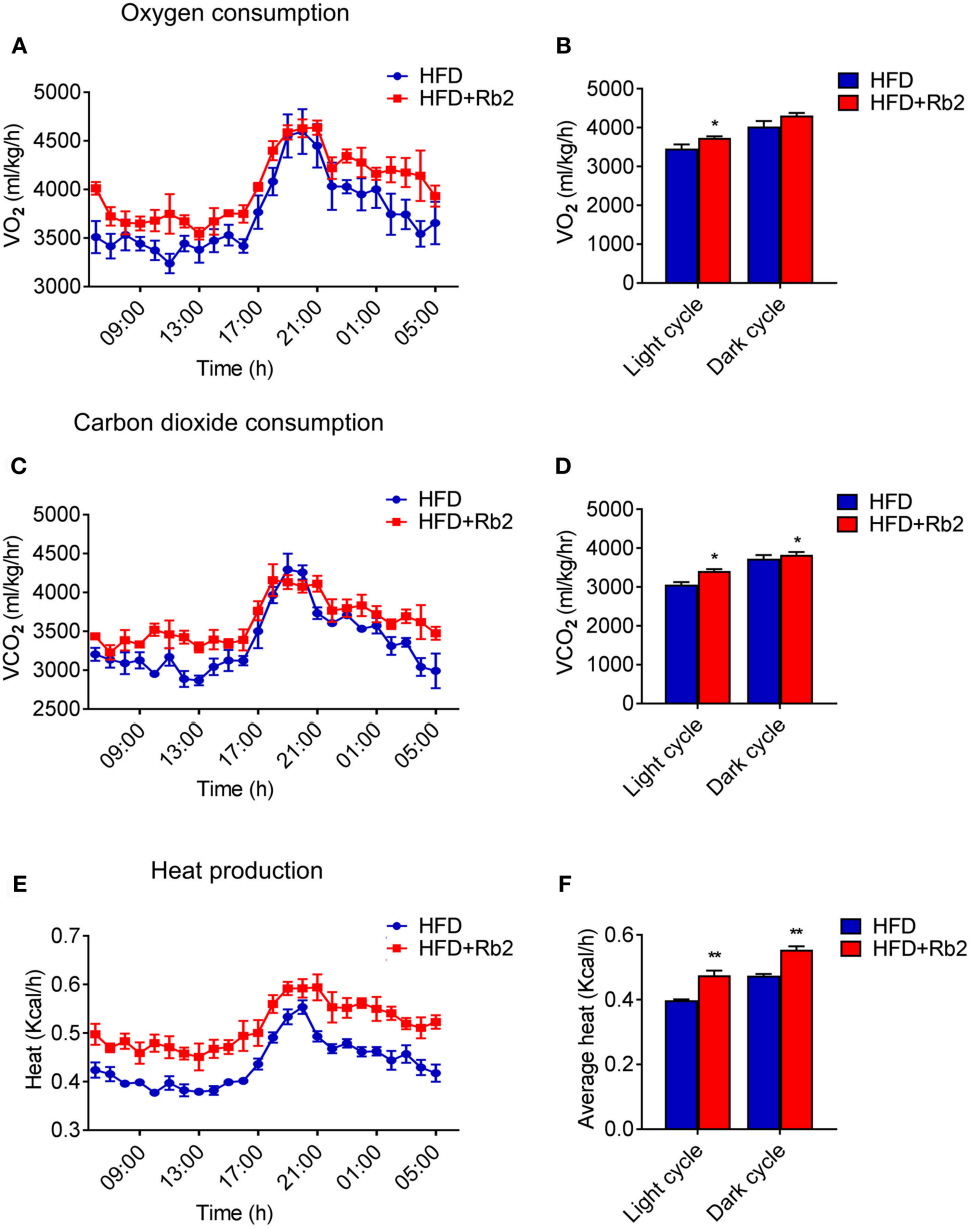
Rb2 treatment increased energy expenditure in DIO mice. **(A–F)** Energy expenditure was evaluated by measurement of oxygen consumption (V_O2_) **(A)** carbon dioxide release (V_CO2_) **(C)** heat production **(E)** over a 24 h period in DIO mice after 10 days of PBS or Rb2 treatment. The average numbers calculated as day and night were shown in **(B,D,F)**. *N* = 6 per group. Data are presented as mean ± SEM and ^*^P < 0.05, ^**^P < 0.01 compared to HFD group.

In order to better understand the mechanism of Rb2 in ameliorating obesity and improving insulin sensitivity, we systematically analyzed the actions of Rb2 on energy expenditure using comprehensive lab animal monitoring system (CLAMS). The Rb2 treated mice showed markedly higher oxygen consumption and carbon dioxide production rates through a 12 h light/dark cycle than the control group (Figures 2A–D). Besides, DIO mice received Rb2 exhibited a significant increase in the whole-body energy expenditure shown as heat production (Figures 2E,F). These results suggested that Rb2 increased energy expenditure in DIO mice.

### Rb2 Treatment Reduced Adiposity and Induced Brown Fat Gene Programs in Adipose Tissues

**Figure 3:**
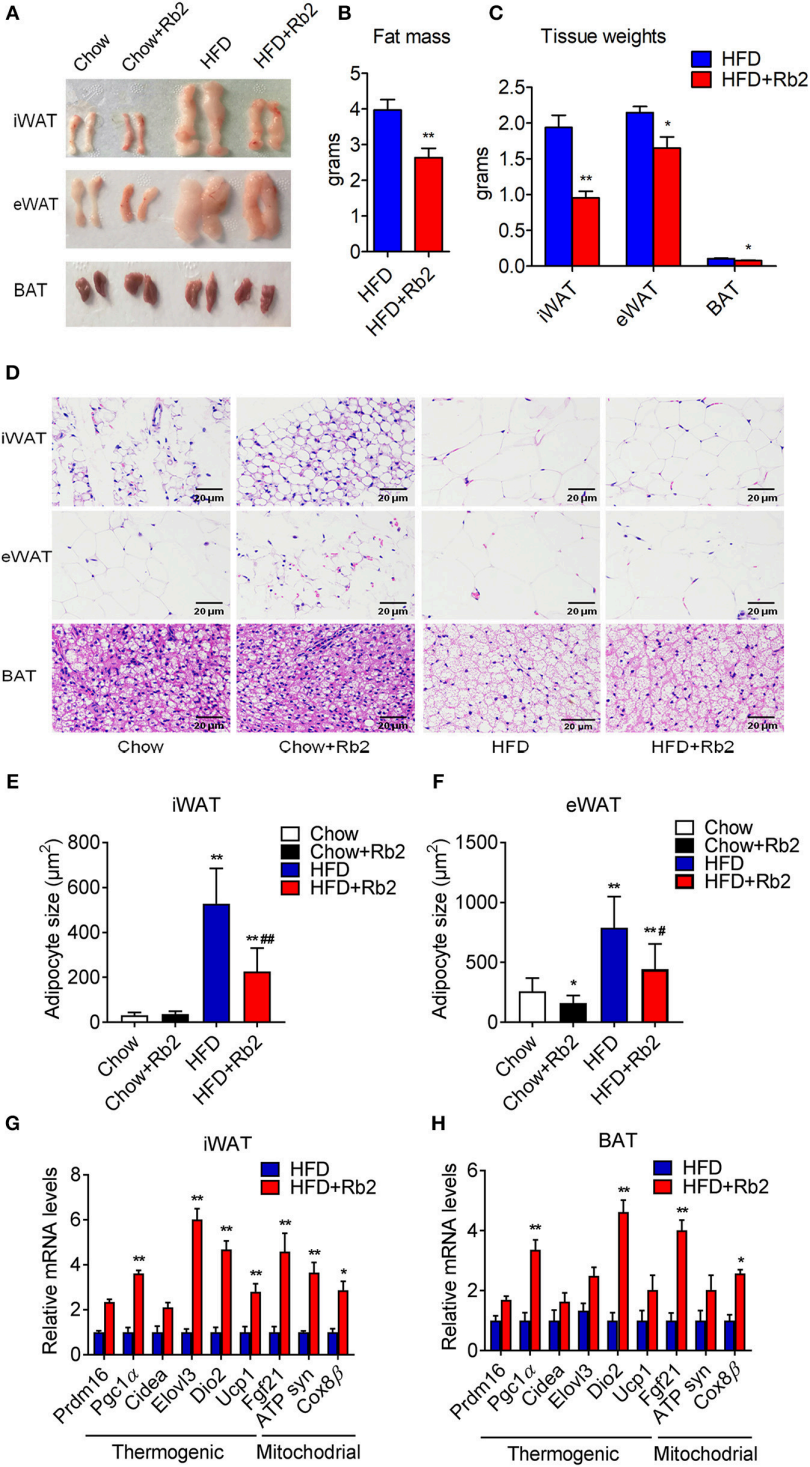
Rb2 treatment reduced adiposity and induced brown fat gene programs in adipose tissues. **(A–C)** Representive photographs, fat mass and tissue weight of iWAT, eWAT, and BAT of DIO mice treated with Rb2 and PBS for 10 days. **(D–F)** Representative hematoxylin and eosin staining from iWAT, eWAT, and BAT sections and quantifications of adipocytes sizes of white fat. **(G,H)** gene expression analysis of thermogenic and mitochondrial genes in iWAT and BAT. *N* = 6 per group. Data are presented as mean ± SEM, ^*^P < 0.05, ^**^P < 0.01 compared to chow group and ^#^*P* < 0.05, ^##^*P* < 0.01 compared to HFD group.

To further explore the effect of Rb2 on adiposity, we closely examined the adipose tissue morphology and molecular signatures. We found that Rb2 treatment significantly decreased fat mass and weights of iWAT, eWAT, and BAT in DIO mice compared with control groups (Figures 3A–C). Consistently, Rb2 treated mice showed smaller adipocytes sizes as shown by histological analysis (Figures 3D–F). Moreover, we performed gene expression analysis on adipose tissues of DIO mice treated with or without Rb2. As shown in Figures 3G,H, Figure S2, several thermogenic and mitochondrial genes were significantly increased in iWAT and BAT of Rb2 treated mice with HFD while mildly elevated in mice with chow diet.

### Rb2 Treatment Enhanced Adaptive Thermogenesis and Induced Browning of White Fat

**Figure 4:**
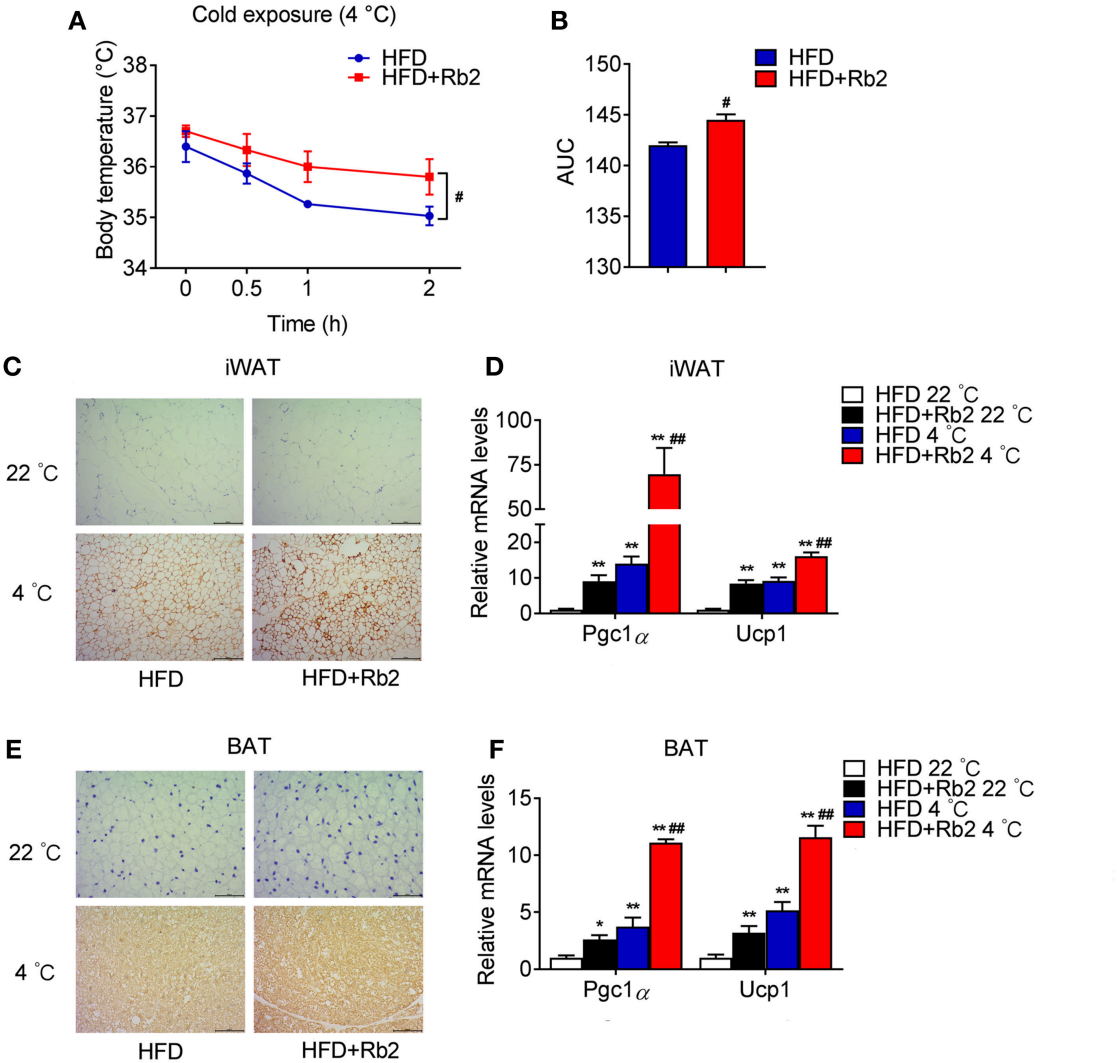
Rb2 treatment increased adaptive thermogenesis. **(A,B)** Rectal body temperature and related AUC of DIO mice treated with or without Rb2 during cold tolerance test. **(C–F)** Immunohistochemistry for UCP1 staining and gene expression of Pgc1α and Ucp1 in iWAT and BAT of DIO mice treated with or without Rb2 under cold stimuli for 24 h. *N* = 6 per group. Data are presented as mean ± SEM and ^#^*P* < 0.05 compared to HFD group in **(A,B)**, ^*^P < 0.05, ^**^P < 0.01 compared to HFD 22°C group and ^#^*P* < 0.05, ^##^*P* < 0.01 compared to HFD 4°C group in **(D,F)**.

Cold exposure is known to induce adaptive thermogenesis, brown fat activation, and browning of white adipose tissues. Thus, we performed cold tolerance tests to assess the thermogenic capacity of mice treated with Rb2 and found that Rb2 treatment significantly ameliorated the cold-induced reduction in rectal temperatures (Figures 4A,B). In addition, we found profound morphological transformation toward a brown fat phenotype and increased UCP1 staining in iWAT of Rb2 treated mice (Figures 4C,E), accompanied with the synergistic effects of Rb2 and cold in the induction of Pgc1α and Ucp1 in both iWAT and BAT (Figures 4D,F). These results suggested that Rb2 treatment enhanced adaptive thermogenesis and induced browning of white fat.

### Rb2 Treatment Induced Browning of White Adipocytes

**Figure 5:**
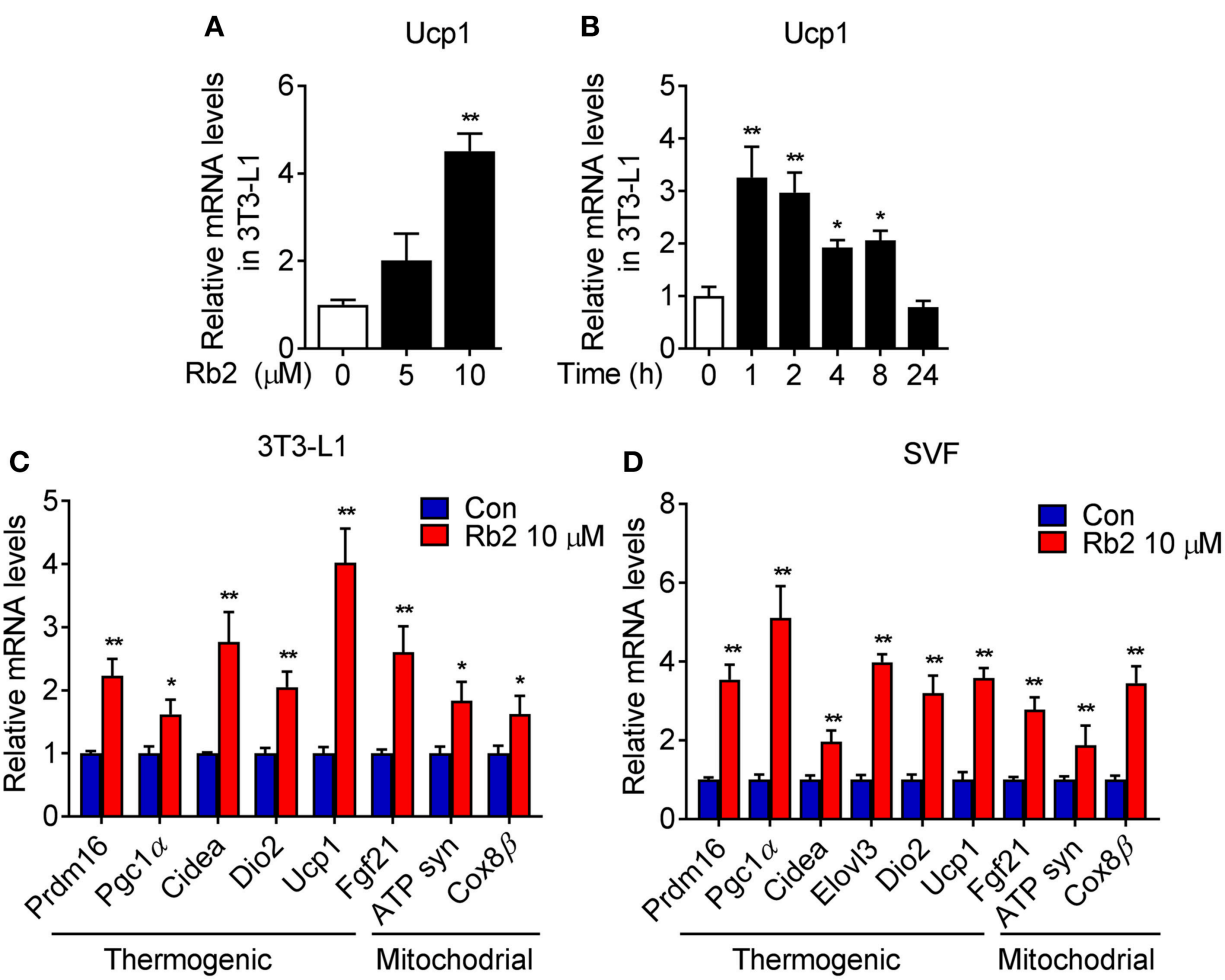
Rb2 treatment induced brown gene programs in white adipocytes. **(A,B)** mRNA levels of Ucp1 in 3T3-L1 cells treated with or without Rb2 in dose and time dependent manner. **(C,D)** mRNA levels of thermogenic and mitochondrial genes in 3T3-L1 adipocytes and differentiated adipocytes from primary iWAT SVF. *N* = 6 per group. Data are presented as mean ± SEM and ^*^P < 0.05, ^**^P < 0.01 compared to control group.

To further examine the cell autonomous effects of Rb2 on adipocytes, we examined the Rb2 treatment on differentiated 3T3-L1 and C3H10T1/2 adipocytes and differentiated SVF from iWAT of mice. As shown in Figures 5A,B, Rb2 dose- and time-dependently induced Ucp1 transcription in 3T3-L1 adipocytes. Furthermore, treatment of Rb2 in adipocytes strongly induced thermogenic and mitochondrial genes, including Pgc1α and Ucp1 levels in 3T3-L1 and C3H10T1/2 adipocytes, and also differentiated SVF from iWAT of mice (Figures 5C,D, Figure S3). Therefore, the data suggested the effect of Rb2 on browning of white adipocytes.

### AMPK Signaling Was Dispensable for the Effects of Rb2 on Browning of White Adipose Tissues and White Adipocytes

**Figure 6:**
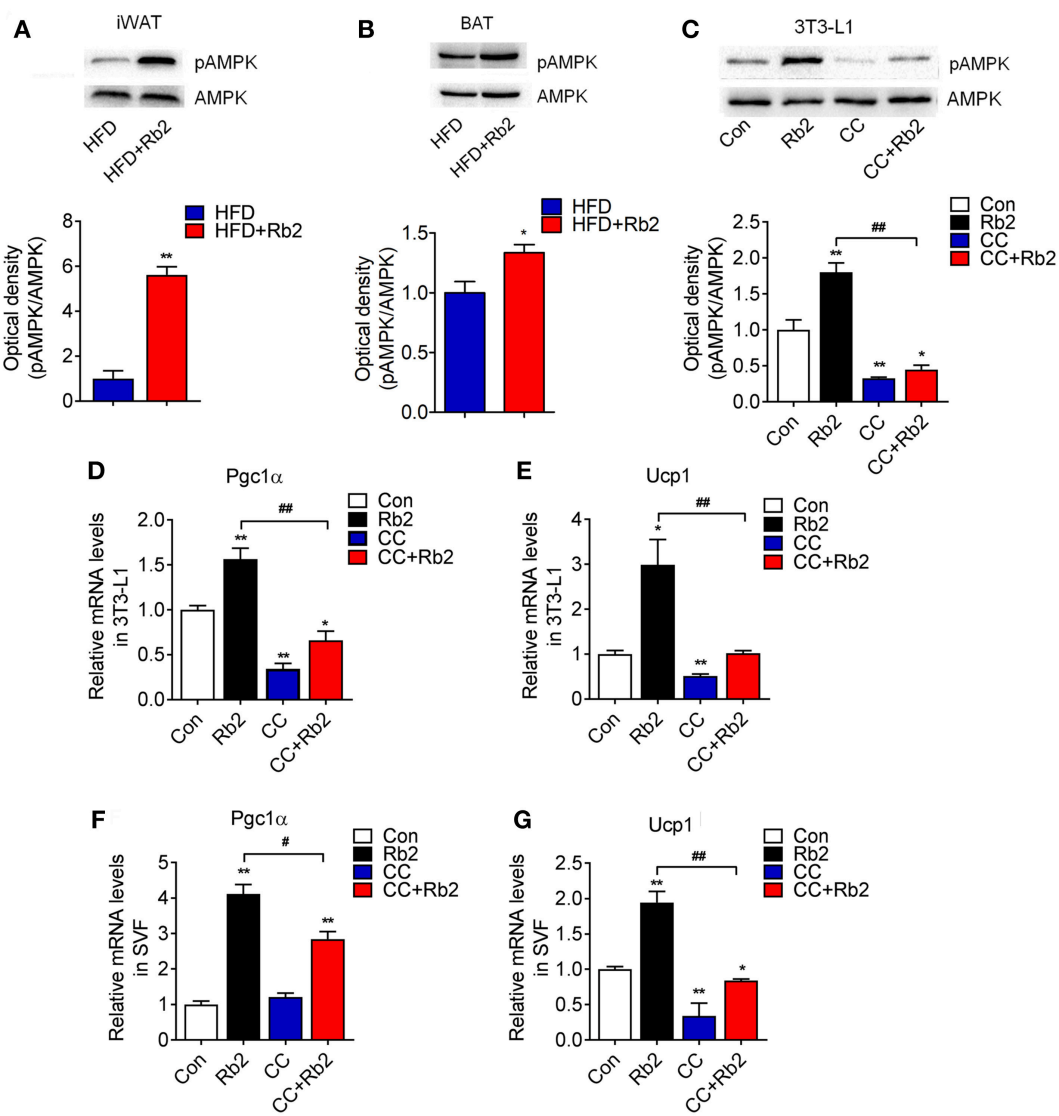
AMPK signaling pathway was dispensable for the beneficial effects of Rb2 in browning. **(A,B)** Phosphorylation and total protein levels of AMPK in response to Rb2 treatment in the iWAT and BAT of DIO mice. **(C)** Phosphorylation and total AMPK levels in 3T3-L1 adipocytes treated with or without Rb2 in the absence or presence of compound C (10 μM). mRNA levels of Pgc1α and Ucp1 in 3T3-L1 adipocytes **(D,E)** and differentiated adipocytes from primary iWAT SVF **(F,G)** treated with or without Rb2 in the absence or presence of compound C. Data are presented as mean ± SEM, ^*^P < 0.05, ^**^P < 0.01 compared to control group and ^#^*P* < 0.05, ^##^*P* < 0.01 compared to Rb2 group.

AMPK signaling has been shown to play a critical role in energy homeostasis and obesity, which is associated with reduced AMPK activity in both WAT and BAT (28–30). Therefore, we examined AMPK signaling in Rb2 treated mice and adipocytes. Western-blot analysis showed that Rb2 supplementation increased phosphorylation of AMPK in iWAT and BAT of both chow diet-fed and DIO mice, which suggested the activation of AMPK signaling in these adipose tissues (Figures 6A,B, Figure S4). Consistently, Rb2 administration enhanced the phosphorylation of AMPK, as well as its downstream thermogenic genes Pgc1α and Ucp1 in differentiated adipocytes (Figures 6C–E). Furthermore, when using AMPK inhibitor compound C, the induction of AMPK phosphorylation, as well as Pgc1α and Ucp1 mRNA levels were diminished in differentiated adipocytes (Figures 6C–E) and SVF from iWAT of mice (Figures 6F,G), suggesting that AMPK was dispensable for the beneficial effects of Rb2.

## Discussion

Obesity is characterized by the reduced metabolic activity of brown and beige fat (31, 32), which suggests that therapies to restore brown/beige fat functionality may be effective to overcome obesity. To date, researches have shown that monomers of TCM, such as celastrol and berberine, play important roles in the remodeling of adipose tissue and prevention of obesity, suggesting the vast choices of TCM in the frontline combating obesity. Ginsenosides, as a class of natural product steroid glycosides and triterpene saponins, are able to improve lipid and glucose metabolism and reduce obesity (33). However, the molecular mechanisms have not been fully understood. Previous reports on the *in vivo* metabolic effects of ginsenosides focused mainly on liver, skeleton muscle and found that in liver, Rg1, Rg3, Rg5, and Rb2 prevented hepatic steatosis with AMPK as their possible common target (15, 18, 20, 34). Besides, Ginsenosides Rb1, Rg1, Rg5, and Re target skeleton muscle for enhancing insulin sensitivity (35–38) and ginsenosides Rb1 may target the central nervous system in obese mice to improve leptin sensitivity (39). While the effects of Ginsenosides on adipocytes are mainly recorded in the 3T3-L1 system, the present study focusing on the brown/beige fat functionality provides both *in vitro* and *in vivo* evidences that Rb2, one of the major effective monomers in ginsenosides, could reduce body weight and improve glycemic and lipid metabolism by activating brown fat functionality, promoting white adipose browning and increasing energy expenditure. Whether other ginsenosides also contribute to the browning process still needs further investigation. In our study, an appropriate dose of Rb2 supplement could be considered as a possible approach to reduce the risk of diet-induced obesity and insulin intolerance.

A complication of obesity is hyperinsulinemia. Specifically, HFD feeding can cause primary hyperinsulinemia, develop insulin resistance, and increase blood glucose levels (40). It is well-established that metabolic organs including pancreas, liver, skeleton muscle and adipose tissues are involved in glycemic control and all contribute to insulin sensitivity (41). Although we found that the Rb2 treatment led to brown fat activation and browning of white fat, it is not clear which is the major and primary organ responsible for improving insulin sensitivity observed in Rb2 treated mice, especially considering previous reports recording the beneficial effects of Rb2 in improving hepatic steatosis. Future studies with euglycemic clamp would be more informative to distinguish the role of Rb2 in glycemic control.

Obesity develops when the energy intake exceeds the energy expenditure, treatment for obesity must either reduce energy intake or increase energy expenditure, or have an effect on both at the same time. In our study, the food intake in mice treated with Rb2 showed no significant differences from the control group. Our findings indicated that Rb2 reduced the adipocytes area in DIO mice and eWAT of chow mice, but it had no effect on weight loss compared to the control group, which was also observed in other studies (42)—it further confirmed the safeness of Rb2 in chow animals. Several researches have shown that Rb2 lowered cholesterol and triacylglycerol levels in 3T3-L1 (22). This might be another important mechanism of Rb2 limits weight gain.

AMPK is a highly conserved master regulator of metabolism at both the cellular and organismal levels. AMPK is actively involved in multiple physiological and pathological processes and regulates food intake, glucose homeostasis (43), hepatic glucose production, lipid metabolism (44), energy homeostasis, body weight (45), browning of WAT, and BAT thermogenesis (46). Specifically, AMPK extensively regulates thermogenesis by modulating the sympathetic nervous system. Genetic evidence with the specific deletion of AMPK in adipocytes led to defects in BAT mitochondrial structure, function, and reduced oxidative metabolism in response to cold exposure or β-adrenergic stimulation. Besides, loss of adipocytic AMPK exacerbated the development of hepatic steatosis and insulin resistance (47). Thus, AMPK agonists such as resveratrol and A-769662 have been applied to alleviate obesity and metabolic disorders. To investigate whether Rb2 inducing brown gene programs in adipocytes was dependent on AMPK, we inhibited the activity of AMPK via compound C in the presence or absence of Rb2. Our results showed that supplement with compound C significantly suppressed the expressions of PGC-1α and UCP1, suggesting that AMPK was at least partially responsible for the effects of Rb2 in adipocytes. Compound C is one of the most widely used compounds as AMPK inhibitor to assess AMPK signaling pathway alternations, but it has limitations. And more analysis on Rb2 mediated signaling pathways may be applied to fully elucidate the mechanisms of Rb2 in adipocytes.

In summary, the present study with both *in vivo and in vitro* evidence highlighted the beneficial effects of Rb2 in treating diet-induced obesity and insulin resistance. Our results demonstrated that Rb2 activated brown fat functionality, induced browning of white fat, and consequently increased thermogenesis and energy expenditure. We further showed that the protective effect of Rb2 against obesity might be potentially modulated by inducing AMPK phosphorylation and its downstream thermogenic and mitochondrial genes. These findings provide rationality to study the effects of other ginsenosides on the brown/beige fat functionality, and suggest the importance of potentially applying Rb2 to the treatment of obesity and metabolic functions.

## Data Availability

Publicly available datasets were analyzed in this study. This data can be found online at https://pan.baidu.com/s/1qMWWmEX-OJEdXTIcEBQ5tg.

## Author Contributions

XG conceived and carried out the experiments. YH and YL conceived the experiments and analyzed data. All the authors have significantly contributed to this work. Other authors were involved in writing the paper and searching literature.

### Conflict of Interest Statement

The authors declare that the research was conducted in the absence of any commercial or financial relationships that could be construed as a potential conflict of interest.

## Supplementary Material

Figure S1 The effects of Rb2 treatment in chow diet-fed mice. **(A,B)** Body weights of chow diet and high fat diet-fed mice for 9 weeks. **(C)** Body weights of chow diet-fed mice treated with or without Rb2 for 10 days. Average of food intakes (g) of DIO mice during the Rb2 treatment **(D)**. **(E–H)** GTT **(E)** and ITT **(G)** analysis of chow diet-fed mice treated with or without Rb2 for 10 days. Area under the curve (AUC) of GTT and ITT was also shown as **(F,H)**. *N* = 6 per group. Data are presented as mean ± SEM.

Figure S2 The mRNA levels of brown gene programs in iWAT and BAT of chow diet-fed mice treated with or without Rb2. **(A,B)** mRNA levels of thermogenic and mitochondrial genes in iWAT **(A)** and BAT **(B)** of chow diet-fed mice treated with or without Rb2. *N* = 6 per group. Data are presented as mean ± SEM and ^**^*P* < 0.01 compared to control group.

Figure S3 The mRNA levels of Pgc1α and Ucp1 in differentiated C3H10T1/2 adipocytes treated with or without Rb2. Data are presented as mean ± SEM, ^*^*P* < 0.05 compared to control group.

Figure S4 p-AMPK and AMPK protein levels in iWAT and BAT of chow diet-fed mice treated with or without Rb2. **(A,B)** Phosphorylation and total protein levels of AMPK in response to Rb2 treatment in the iWAT and BAT of chow diet-fed mice. Data are presented as mean ± SEM, ^*^*P* < 0.05, ^**^*P* < 0.01 compared to control group.
