# Supplementary figures and images for: Ginsenoside Rb2 Alleviates Obesity by Activation of Brown Fat and Induction of Browning of White Fat
Source: Front Endocrinol (Lausanne). 2019 Mar 15;10:153. doi: 10.3389/fendo.2019.00153 (PMC6428988; doi:10.3389/fendo.2019.00153)

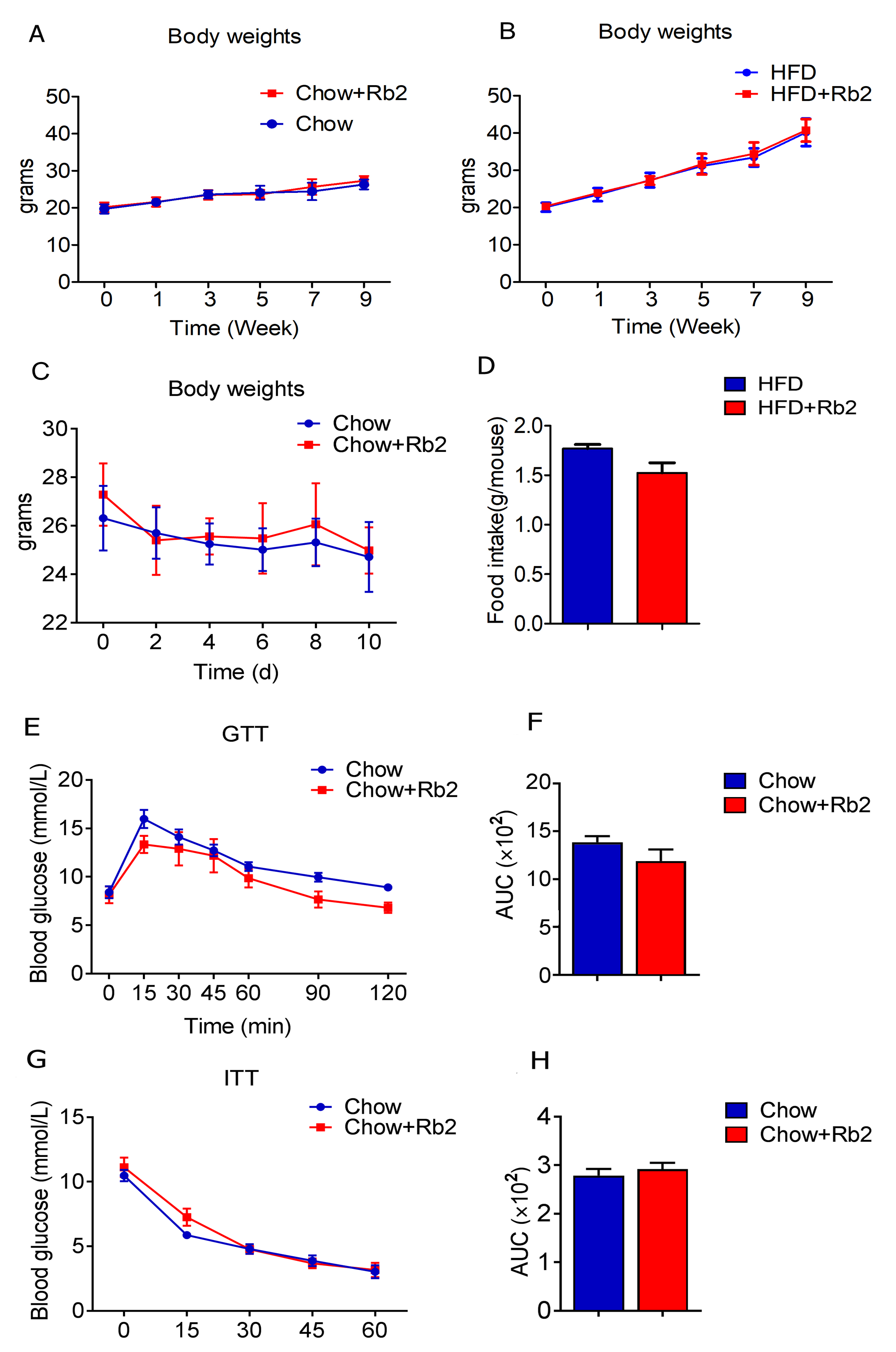

Supplement: Figure S1 — The effects of Rb2 treatment in chow diet-fed mice. (A,B) Body weights of chow diet and high fat diet-fed mice for 9 weeks. (C) Body weights of chow diet-fed mice treated with or without Rb2 for 10 days. Average of food intakes (g) of DIO mice during the Rb2 treatment (D). (E–H) GTT (E) and ITT (G) analysis of chow diet-fed mice treated with or without Rb2 for 10 days. Area under the curve (AUC) of GTT and ITT was also shown as (F,H). N = 6 per group. Data are presented as mean ± SEM. [file Image_1.TIF]

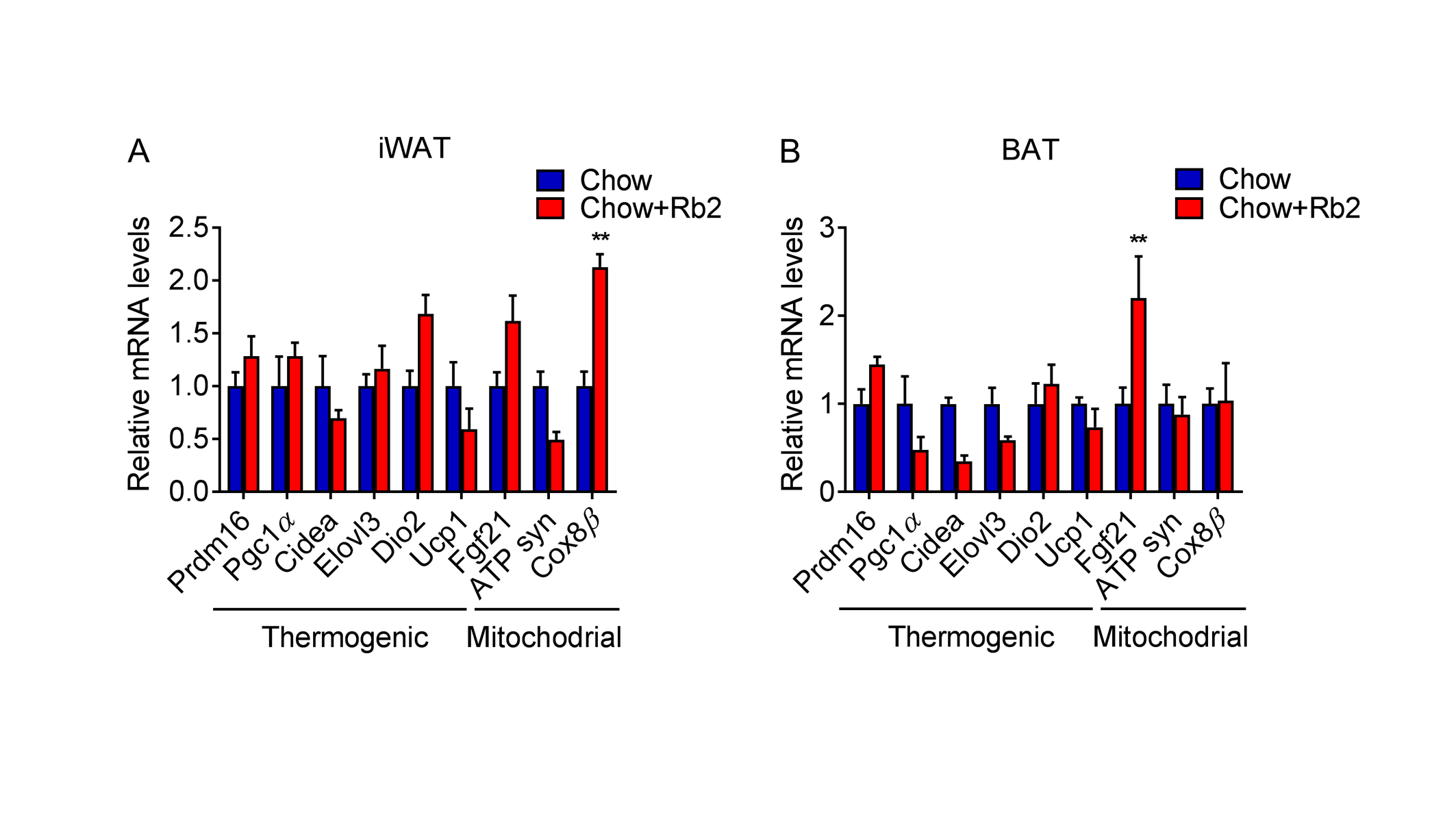

Supplement: Figure S2 — The mRNA levels of brown gene programs in iWAT and BAT of chow diet-fed mice treated with or without Rb2. (A,B) mRNA levels of thermogenic and mitochondrial genes in iWAT (A) and BAT (B) of chow diet-fed mice treated with or without Rb2. N = 6 per group. Data are presented as mean ± SEM and **P < 0.01 compared to control group. [file Image_2.TIF]

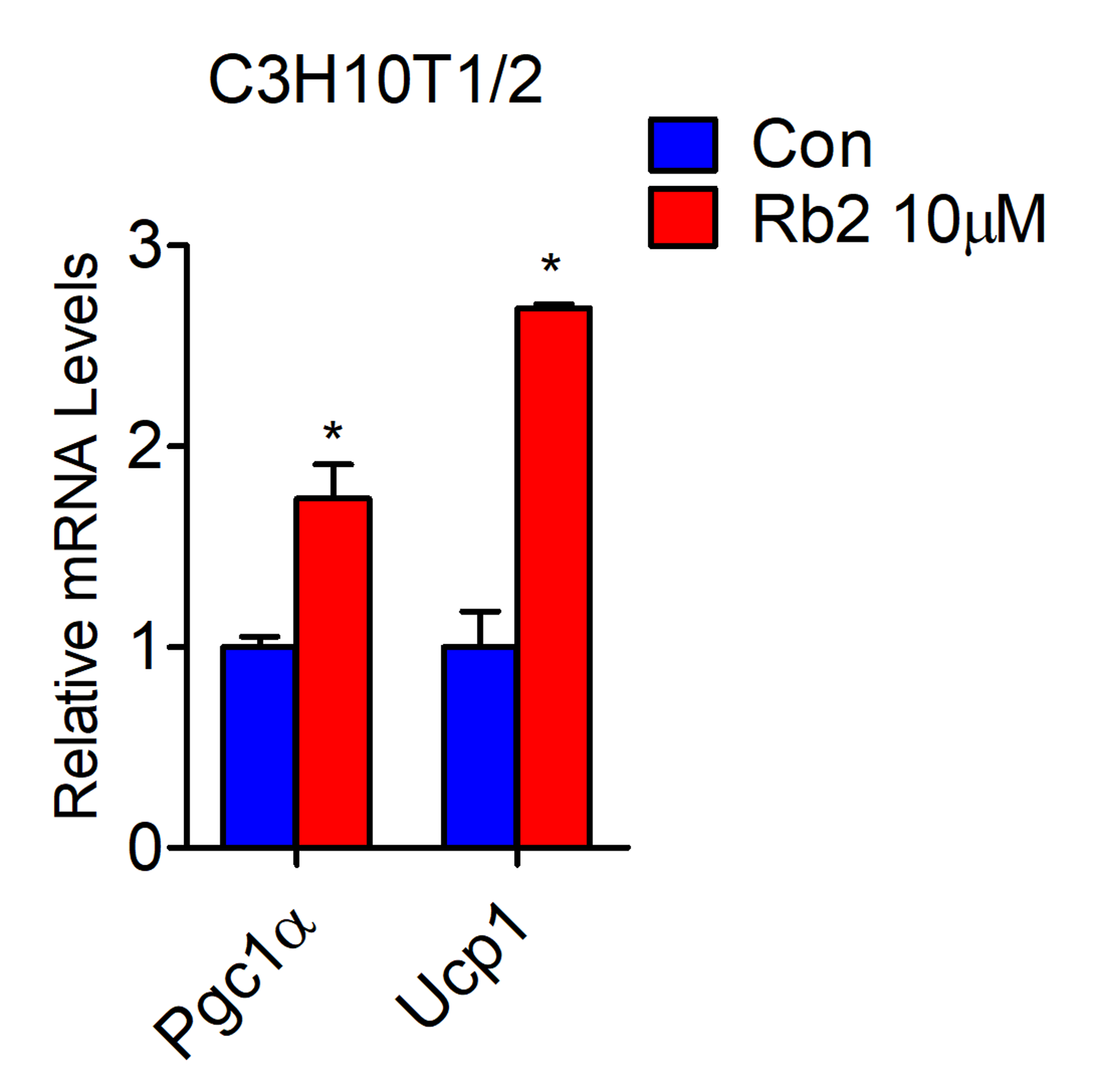

Supplement: Figure S3 — The mRNA levels of Pgc1α and Ucp1 in differentiated C3H10T1/2 adipocytes treated with or without Rb2. Data are presented as mean ± SEM, *P < 0.05 compared to control group. [file Image_3.TIF]

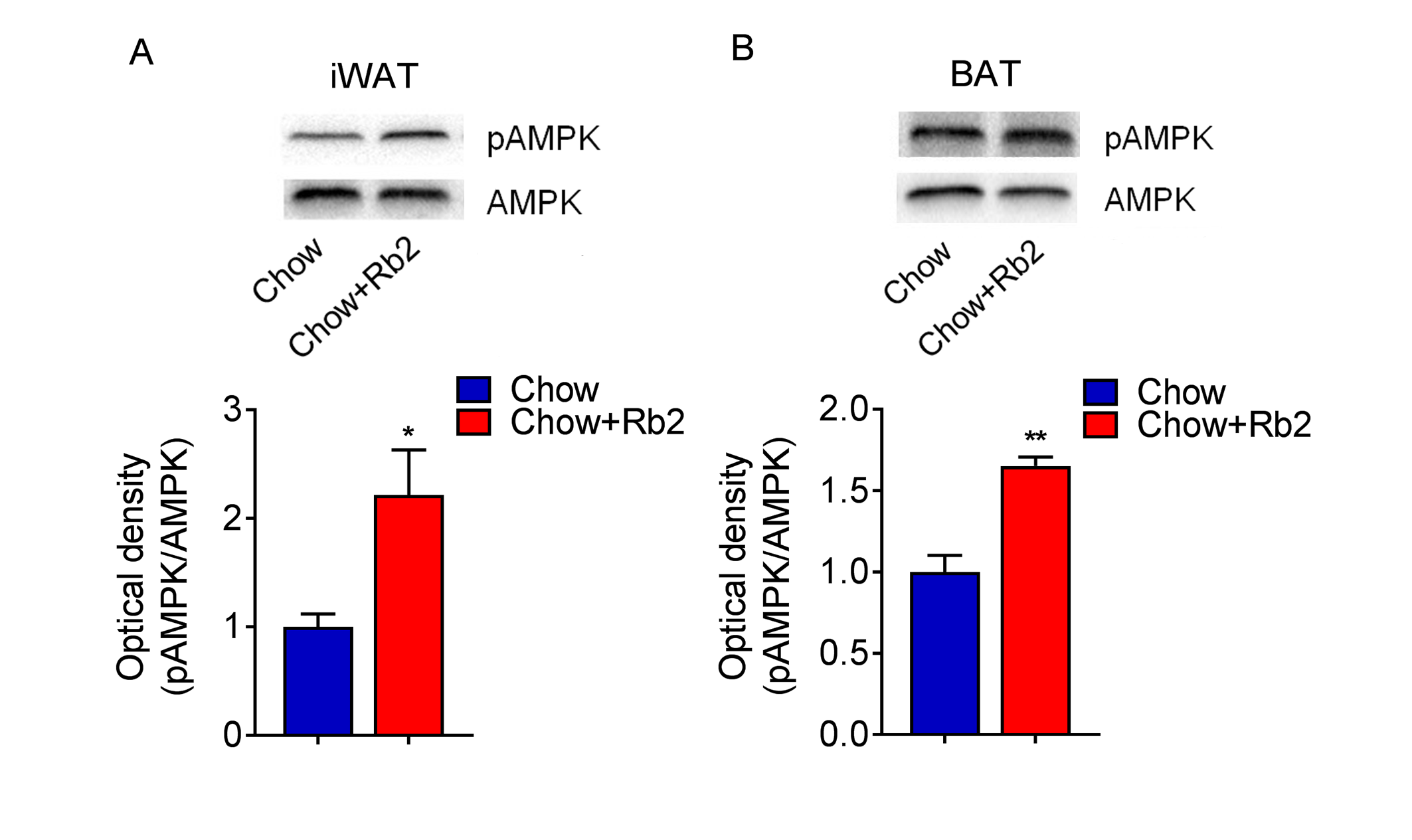

Supplement: Figure S4 — p-AMPK and AMPK protein levels in iWAT and BAT of chow diet-fed mice treated with or without Rb2. (A,B) Phosphorylation and total protein levels of AMPK in response to Rb2 treatment in the iWAT and BAT of chow diet-fed mice. Data are presented as mean ± SEM, *P < 0.05, **P < 0.01 compared to control group. [file Image_4.TIF]
